# Do Lower Calorie or Lower Fat Foods Have More Sodium Than Their Regular Counterparts?

**DOI:** 10.3390/nu8080511

**Published:** 2016-08-19

**Authors:** Katherine A. John, Joyce Maalouf, Christina B. Barsness, Keming Yuan, Mary E. Cogswell, Janelle P. Gunn

**Affiliations:** 1 Epidemiology and Surveillance Branch, Division for Heart Disease and Stroke Prevention, National Center for Chronic Disease Prevention and Health Promotion, Centers for Disease Control and Prevention, 4770 Buford Hwy NE, Atlanta, GA 30341, USA; vjh6@cdc.gov (J.M.); vrm4@cdc.gov (K.Y.); mec0@cdc.gov (M.E.C.); 2 IHRC, Inc., 2 Ravinia Dr Ste 1750, Atlanta, GA 30346, USA; 3 Department of Public Health, St. Catherine University, 2004 Randolph Avenue, Saint Paul, MN 55105, USA; cdblissbarsness@stkate.edu; 4 Division of Nutrition, Physical Activity, and Obesity, National Center for Chronic Disease Prevention and Health Promotion, Centers for Disease Control and Prevention, 4770 Buford Hwy NE, Atlanta, GA 30341, USA; bfy2@cdc.gov

**Keywords:** sodium, lower fat, lower calorie, food products, nutrient information

## Abstract

The objective of this study was to compare the sodium content of a regular food and its lower calorie/fat counterpart. Four food categories, among the top 20 contributing the most sodium to the US diet, met the criteria of having the most matches between regular foods and their lower calorie/fat counterparts. A protocol was used to search websites to create a list of “matches”, a regular and comparable lower calorie/fat food(s) under each brand. Nutrient information was recorded and analyzed for matches. In total, 283 matches were identified across four food categories: savory snacks (*N* = 44), cheese (*N* = 105), salad dressings (*N* = 90), and soups (*N* = 44). As expected, foods modified from their regular versions had significantly reduced average fat (total fat and saturated fat) and caloric profiles. Mean sodium content among modified salad dressings and cheeses was on average 8%–12% higher, while sodium content did not change with modification of savory snacks. Modified soups had significantly lower mean sodium content than their regular versions (28%–38%). Consumers trying to maintain a healthy diet should consider that sodium content may vary in foods modified to be lower in calories/fat.

## 1. Introduction

Roughly half of all American adults have one or more preventable chronic diseases, many of which are related to poor dietary patterns [1]. Currently, the US population does not meet dietary recommendations [1]. Evidence suggests that the diet of the US population contains excess amounts of sodium, saturated fats, refined grains, sugar, and calories, whereas fruits, vegetables, and whole grains are under consumed [1]. Nutrition messages, relaying characteristics and examples of healthy food choices, can be effective in communicating to the public the importance of nutrition on health [2]. The majority of US adult consumers are aware of the benefits of maintaining a healthy diet. Nine out of 10 US adult consumers reported thinking about the healthfulness of the ingredients in the foods and beverages they consume (48% thought “a lot”, whereas 44% “a little”), while about 50% reported that they were specifically trying to avoid calories, fats/oils, and sodium in food products [3]. Despite awareness, consumers are challenged to maintain a healthy eating pattern, in part due to the abundance and convenience of calorie and sodium dense foods [1,4,5].

Strategies manufacturers have used to assist consumers to make more healthful choices include: reformulating products to decrease nutrients of concern, portion-controlled packaging, and front-of-package nutrition-related information systems [4,6,7,8,9]. New nutrition labeling also may help consumers by emphasizing calories and serving size to reflect typical consumer consumption in one sitting, shifting of the percent Daily Value (DV), and notification of the inclusion of nutrients of public health interest such as added sugars and potassium [10]. Nutrient content claims promoting the modification of an ingredient, are appearing more often in the food supply and may assist consumers in identifying foods that are lower in specific nutrients to improve the nutritional quality of their diet [7,11]. Manufacturers may choose to promote nutrient content claims as a way to satisfy consumer preferences for healthier products and to differentiate their products from competitors [11]. In 2009, U.S. food sales of products with reduced calorie claims accounted for $12 billion and fat claims for $46 billion [12]. Given the availability of packaged foods with nutrient claims, the IOM’s recommendation to expand monitoring of packaged food labels, and concern that as one aspect of a product is modified the sodium content may be increasing, our objective was to compare the sodium content of a regular food product with the matching lower calorie or lower fat counterpart [13,14]. We hypothesized that the modification or reformulation of regular foods to their lower in calorie or fat match, by brand, would result in the addition of salt (sodium chloride) to maintain texture or taste.

## 2. Materials and Methods

Selection of food categories was conducted using the CDC’s packaged food database which contains Nielsen point-of-sales data and Gladson manufacturer and product-level nutrition information on roughly 8000 packaged food items from 2009 [5]. A detailed description of how this database was created is provided elsewhere [5]. The packaged food database consisted of the top 20 USDA food categories shown to contribute the most sodium to the U.S. diet, expanded from a published list of 10 categories [15]. The food categories were evaluated for relevance to this analysis using 3 separate database searches of item name, description, and product details and the following terms regulated by the FDA: “free”, “zero”, “reduced”, “less”, “low”, “lower”, “light”, “lite”, “lean”, or “little” paired with calorie(s) or fat [8]. For a manufacturer to use a claim, the FDA food labeling guide requires indication of claims based on nutrient specific definitions. All reduced/less/light nutrient claims are based on proportional reductions in the nutrient content compared with the same serving size of a matching regular food while free/low nutrient claims are based on the amount of nutrient in the food, e.g., fat-free foods have to have less than 0.5 g per Reference Amounts Customarily Consumed (RACC) and per labeled serving [8]. Exclusion criteria for this analysis included the reference of sodium in either the item name, description, or product details. USDA food categories using the above search, were reviewed using company and manufacturer websites and ranked based on the number of qualifying low calorie or low fat and regular calorie/fat matched brand foods. Food categories that did not have a regular calorie or regular fat equivalent to a lower calorie or lower fat food match within the same brand, were excluded. The four food categories with the highest number of matches were selected for further analysis: savory snacks, cheese, salad dressing, and soup (Supplementary Materials, Figure S1).

Product information, based on matches between regular and reduced calorie/fat foods, was collected using data pulled between June 2013 and January 2014. Nielsen national sales data were used to identify the top 10 brands, that when totaled together comprised at least 50% of the market share within the food category. For each food category, foods sold under each of the 10 brands were identified through manufacturer and other reliable websites (Supplementary Materials, Figure S2). On these sites, low calorie and low fat foods in each brand were identified by reviewing the title or package for the following terms: “baked”, “calorie free”, “fat free”, “less calories”, “less fat”, “light”, “lite”, “low calorie”, “low fat”, “reduced calorie”, “reduced fat”, “zero calorie”, “zero fat”, “low fat, low in fat, or low in saturated fat”, and reduced calorie portion packaging and foods that advertise lower numeric caloric amounts, for example “100 calorie packs” or “right bites”. From this list, matches were made between regular foods and their comparable lower calorie or lower fat food within each brand. One regular food could have multiple matches to a “low/reduced” counterpart within the same food item of the same brand; for example, regular “Nacho Cheese Flavored Tortilla Chips” might be matched with three modified food items: (1) Nacho Cheese Reduced Fat Tortilla Chips; (2) Baked Nacho Cheese Flavored Tortilla Chips; and (3) Nacho Cheese Flavored Tortilla Chips–100 Calorie Packs. Low/reduced calorie/fat foods that did not have a regular calorie/fat equivalent match within the same brand were excluded. An image of the Nutrition Facts panel (NFP) was recorded for each product from the manufacturer’s website and nutrient information abstracted into a database. If the nutrient information was unavailable from the manufacturer’s website, other websites were used (Supplementary Materials, Figure S2). Private label (store-brand) products, multipack, and variety pack products were excluded (Supplementary Materials, Figure S3).

Mean nutrients (calories, sodium, total fat, and saturated fat) were calculated for the regular and modified foods. Mean nutrient content was calculated per 100 g of food and per labeled serving size to avoid confounding effects of the reduced calorie portion packaging, for regular and modified foods within each food category. Mean serving sizes (g) were calculated for each food category (Supplementary Materials, Table S1). The difference in nutrient content for each match (regular versus the modified food) was calculated and then averaged over all the matched pairs. A negative value signifies a reduction in the average mean of the nutrient content among the modified foods. The percent change was calculated to determine the extent of the increase or decrease related to modification for each food category. Paired *t*-tests were used to test whether the differences in means of regular vs. modified foods were statistically significant. Multiple comparisons within each food category were conducted, lending the possibility that some significant average differences could be due to chance. To minimize this, a Bonferroni correction was used to establish the probability that a change in nutrient content was statistically significant for a specific food category by dividing 0.05 by the number of comparison within the category (*n* = 8). A *p*-value < 0.00625 was considered statistically significant. In separate analyses, we examined nutrient content for foods with a 25% reduction in labeled calories and foods with a 25% reduction in labeled fat as compared to their regular match. Since nutrient content claims are not required on the package, these items were defined through comparative analysis. A modified food could have both a 25% reduction in labeled fat and calories compared to the regular match and be included in both categories. In our analyses, we assumed that modifications to different versions of a product within the same brand were independent. To account for the possibility that manufacturers may make the same changes to all products within different versions of the same brand, a sensitivity analysis was conducted where each regular product in a brand was randomly matched to only one modified product within the same brand (Supplementary Materials, Table S2).

## 3. Results

Across the four food categories, 214 regular food products were identified as having at least one low calorie/fat (modified) counterpart within the same brand, a total of 283 matches (Table 1). Of the 214 regular products, 57 products matched to more than one modified product within the same brand. Roughly 90% of the modified foods had some decrease in calories and total fat, respectively, while only ~40% decreased in sodium per 100 g and per labeled serving size (Table 1). By food category, 90% of modified (low/reduced, calorie/fat) soups also had some decrease in sodium, followed by 48% of modified savory snacks (Table 1). Mean sodium content per 100 g and per serving was 8%–12% higher for modified cheese and salad dressings, but did not differ with modification of savory snacks (Table 2). Mean sodium content was reduced by 38% per 100 g of food and 33% per serving size for modified soups (Table 2). When a regular product was randomly matched to only one modified product, results were similar, with the exception of mean sodium content of modified salad dressings (Supplementary Materials, Table S2) no longer being statistically significant (<0.00625). Mean sodium content of salad dressings per 100 g of food increased by 7.5% with one randomly chosen modified product (*p* = 0.012) (Supplementary Materials, Table S2) compared with 8.4% (*p* = 0.0017) when regular salad dressing was matched with multiple modified products (Table 2). 

In general, the selected modified foods containing at least 25% fewer labeled calories than their regular match had lower average fat content (Table 3). Soups modified to contain at least 25% less labeled calories than their regular counterparts had 33% less sodium per 100 g and 28% less sodium per labeled serving size (*p*-value < 0.001) (Table 3). Savory snacks with 25% less labeled calories did not differ from their regular counterparts in sodium content (Table 3). Cheese and salad dressings with 25% fewer labeled calories had slightly higher sodium per 100 g and per labeled serving (range: 9%–11%) (Table 3). Foods with at least 25% less labeled fat than their regular counterparts also had reduced calories (percent change: calories range −23% to −53%) (Table 3). Across the four categories examined, modified foods containing 25% less labeled fat than their regular counterparts had 6% higher sodium content per 100 g (*p*-value = 0.001), but did not differ in sodium per serving (Table 3). Soups with 25% less labeled fat also had 29%–32% less sodium per 100 g or per serving (*p*-value < 0.001) (Table 3). In comparison, savory snacks with 25% less labeled fat did not differ in sodium content, whereas, the sodium content of salad dressings and cheeses with 25% less labeled fat were higher per 100 g and per serving (range: 9%–12%) (Table 3).

## 4. Discussion

As expected, among the four food categories evaluated (cheese, salad dressings, savory snacks and soups), modifications of foods to reduce calories or fat was associated with lower calories, total fat, and saturated fat. However, changes in sodium content with the calorie and fat modifications varied by food category. For lower calorie and fat soups, sodium content also was lower, but not for savory snacks, cheese and salad dressings. Our findings reinforce the need for consumers not to equate lower calories and fat as the only factors to consider when looking for a healthy food item. 

This report is subject to several limitations. The food product nutritional data were extracted using nutrition facts panels (NFP), which under FDA labeling regulations allows some rounding and variation from what may be the laboratory measurement in a product [16]. Another limitation is that product and brand data would not reflect manufacturer modifications or product introductions after the data were collected (June 2013–January 2014). Products utilized in this study represent a cross-section of the nutritional composition of the most widely purchased packaged foods sold in US grocery stores in 2009 and may not encompass all modified or regular products within each of the investigated food categories [5]. Finally, here we focused on calorie, fat (total and saturated), and sodium content, however, other product reformulations, e.g., adding more sugar to low-fat products [17] or to low-calorie products [18], also may have health implications and deserves further consideration.

While modified products may contain a nutrient content claim, it is still important for consumers to read the product labels and nutrition facts panel when choosing foods. Evidence shows that individuals tend to categorize food-related information into a good/bad (healthy/unhealthy) dichotomy [19]. Chernev and Chandon suggest that this categorization is influenced not only by the nature of food (fruits are considered “good”, while candy is associated as “bad”), but also by nutrition information communicated through the brand name or claims that describe the food (“light” is often categorized as “healthy”, while “creamy” is associated with “unhealthy”) [19]. Chandon and Wansink indicate that when a nutrient claim is introduced to the consumer, it can lead to a “health halo” or an overgeneralization of the claim to include several other nutrients increasing the overall “healthiness” of the whole product [20,21]. For example, consumers may associate a fat-free product as also lower in sodium or calorie content which would increase the desirability of the product. Consumers may be selecting lower calorie or lower fat products thinking they are making a healthier choice and may be inadvertently increasing their sodium intake. Clinicians, registered dieticians, and public health professionals can help by advising their clients to adopt a healthy eating pattern consistent with recommendations [1]. 

Food technology and reformulations enable manufacturers to reduce undesirable components in certain foods; however, additives are sometimes required to deliver the same taste, texture, and mouth feel consumers desire [6,22]. Some added constituents, such as sodium containing ingredients, are used to increase palatability and to increase product shelf life [1]. Many manufacturers have voluntarily committed to making changes to lower the sodium and calories, among other changes to their product portfolio. Ideally, specific techniques and additives used in the reformulation process will improve the nutritional profile as a whole. Earlier changes to reduce or eliminate trans-fat showed that for many products the saturated fat also decreased, while mono- and poly-saturated fats increased [23]. Continuous efforts to efficiently monitor the labels and to update national nutrient databases of packaged foods would enhance the knowledge about the quality and comprehensiveness of sodium and other nutrient content in the U.S. food supply [13]. Furthermore, food modeling studies may help demonstrate the effects that reformulations (e.g., replacing total fat with sodium) may have on population health.

## 5. Conclusions

Lower calorie or lower fat foods can be higher or lower in sodium, depending on the food category. Health conscious consumers can review the nutrition facts panel for sodium content and compare to similar products.

## Figures and Tables

**Table 1:** Difference in modified foods nutrient content compared to regular foods in each of the four food categories ^1^.

Product Categories	Total # of Products	# of Matches	Proportion (%) of Modified Foods That Decreased in							
			Per 100 g of Food				Per Labeled Serving Size			
			Calories	Sodium	Total Fat	Sat Fat	Calories	Sodium	Total Fat	Sat Fat
Total	497	283	90.1	39.2	92.6	87.6	90.1	37.8	90.5	84.5
Cheese	188	105	92.4	22.9	97.1	95.2	91.4	23.8	93.3	91.4
Salad dressings	161	90	100.0	28.9	100.0	95.6	100.0	24.4	100.0	95.6
Savory Snacks	67	44	81.8	47.7	81.8	84.1	84.1	47.7	79.6	77.3
Soups	81	44	72.7	90.9	77.3	56.8	72.7	88.6	75.0	52.3

Sat, saturated. ^1^ A regular food product may be matched to more than one modified food.

**Table 2:** Mean calorie, sodium, total fat, and saturated fat by food category for regular and modified foods.

	Per 100 g of Food				Per Labeled Serving Size			
	Mean (SE) Calories (kcal)	Mean (SE) Sodium (mg)	Mean (SE) Total Fat (g)	Mean (SE) Sat. Fat (g)	Mean (SE) Calories (kcal)	Mean (SE) Sodium (mg)	Mean (SE) Total Fat (g)	Mean (SE) Sat. Fat (g)
**Cheese**								
Regular (*n* = 83)	347.3 (8.1)	698.6 (34.8)	27.4 (0.8)	17.0 (0.5)	91.3 (2.2)	174.3 (7.1)	7.2 (0.2)	4.5 (0.1)
Modified (*n* = 105)	257.5 (7.8)	762.1 (38.9)	16.0 (0.8)	9.8 (0.5)	69.5 (2.2)	191.2 (7.9)	4.3 (0.2)	2.6 (0.1)
Average Difference ^1^	−78.2 (4.9)	72.9 (11.3)	−10.3 (0.6)	−6.5 (0.4)	−20.8 (1.8)	20.4 (3.5)	−2.8 (0.2)	−1.7 (0.1)
Percent Change ^2^	−22.5	10.4	−37.6	−38.2	−22.8	11.7	−38.9	−37.8
*p*-value ^3^	**<0.001**	**<0.001**	**<0.001**	**<0.001**	**<0.001**	**<0.001**	**<0.001**	**<0.001**
**Salad Dressings**								
Regular (*n* = 71)	422.3 (12.1)	889.8 (29.3)	40.3 (1.5)	6.0 (0.3)	127.5 (3.8)	269.6 (9.6)	12.1 (0.4)	1.8 (0.1)
Modified (*n* = 90)	192.1 (7.3)	971.1 (25.6)	12.1 (1.0)	1.8 (0.2) ^4^	58.9 (2.2)	299.1 (8.0)	3.6 (0.3)	0.5 (0.1) ^4^
Average Difference ^1^	−222.6 (8.6)	74.3 (22.8)	−27.3 (1.1)	−4.1 (0.2)	−66.4 (2.7)	26.9 (7.5)	−8.2 (0.3)	−1.2 (0.1)
Percent Change ^2^	−52.7	8.4	−67.7	−68.3	−52.1	10.0	−67.8	−66.7
*p*-value ^3^	**<0.001**	**0.002**	**<0.001**	**<0.001**	**<0.001**	**<0.001**	**0.001**	**<0.001**
**Savory Snacks**								
Regular (*n* = 23)	533.9 (6.4)	699.5 (45.4)	33.0 (0.8)	7.8 (1.0)	155.2 (1.9)	204.8 (14.6)	9.7 (0.3)	2.4 (0.4)
Modified (*n* = 44)	453.3 (15.7)	645.7 (21.8)	19.7 (1.8)	5.2 (0.8)	130.8 (5.9)	188.6 (9.2)	5.8 (0.6)	1.6 (0.3)
Average Difference ^1^	−81.6 (16.5)	−15.7 (27.8)	−13.5 (1.9)	−3.1 (0.6)	−24.2 (5.7)	−4.1 (9.9)	−3.9 (0.6)	−0.9 (0.2)
Percent Change ^2^	−15.3	−2.2	−40.9	−39.7	−15.6	−2.0	−40.2	−37.5
*p*-value ^3^	**<0.001**	0.580	**<0.001**	**<0.001**	**0.001**	0.684	**<0.001**	**<0.001**
**Soups**								
Regular (*n* = 37)	62.5 (3.3)	428.7 (29.2)	2.0 (0.3)	0.5 (0.1)	124.3 (5.6)	805.7 (15.7)	3.9 (0.5)	1.0 (0.1)
Modified (*n* = 44)	50.2 (2.7)	285.5 (18.6)	1.1 (0.1)	0.3 (0.0) ^5^	98.6 (4.3)	543.4 (24.1)	2.0 (0.1)	0.6 (0.1)
Average Difference ^1^	−14.1 (2.4)	−163.4 (20.2)	−1.2 (0.2)	−0.3 (0.1)	−24.5 (4.0)	−267.0 (27.1)	−2.1 (0.4)	−0.5 (0.1)
Percent Change ^2^	−22.6	−38.1	−60.0	−60.0	−19.7	−33.1	−53.8	−50.0
*p*-value ^3^	**<0.001**	**<0.001**	**<0.001**	**0.001**	**<0.001**	**<0.001**	**<0.001**	**<0.001**

SE = Standard Error; Sat = Saturated. Boldface signifies statistical significance (*p* < 0.00625), Bonferroni adjustment for multiple comparisons of *p* < 0.05. ^1^ The difference in nutrient content for each match was calculated and averaged over all matched pairs (modified-regular). The number of matched pairs for each analysis is equal to the number of modified products. One regular food may have one or more modified food counterparts; ^2^ Percent Change was calculated using the following formula: $\frac{{\text{Mean}}_{\text{Difference}}}{{\text{Mean}}_{\text{Reg}}}*100$; ^3^ *p*-values were determined by paired t-tests between regular and modified foods. Statistical significance is defined as *p*-value < 0.00625; ^4^ One modified salad dressing product was excluded due to lack of information on saturated fat content (*N* = 89); ^5^ SE < 0.05.

**Table 3:** Mean calorie, sodium, total fat, and saturated fat by food category for regular and modified foods of matches where the modified food had at least a 25% reduction in labeled calories and/or fat ^1^.

	Per 100 g of Food				Per Labeled Serving Size			
	Mean (SE) Calories (kcal)	Mean (SE) Sodium (mg)	Mean (SE) Total Fat (g)	Mean (SE) Sat. Fat (g)	Mean (SE) Calories (kcal)	Mean (SE) Sodium (mg)	Mean (SE) Total Fat (g)	Mean (SE) Sat. Fat (g)
**25% Reduction in Labeled Calories**								
**Cheese**								
Regular (*n* = 35)	335.5 (13.0)	715.9 (60.8)	25.7 (1.3)	15.8 (0.8)	90.3 (3.6)	173.9 (11.1)	6.9 (0.4)	4.3 (0.2)
Modified (*n* = 43)	213.8 (12.9)	763.8 (68.0)	12.0 (1.5)	7.2 (0.9)	57.3 (2.9)	185.7 (13.5)	3.1 (0.4)	1.8 (0.2)
Average Difference ^2^	−109.7 (7.8)	80.2 (18.2)	−12.9 (1.1)	−8.1 (0.7)	−34.3 (1.8)	17.7 (5.3)	−3.9 (0.3)	−2.5 (0.2)
Percent Change ^3^	−32.7	11.2	−50.2	−51.3	−38.0	10.2	−56.5	−58.1
*p*-value ^4^	**<0.001**	**<0.001**	**<0.001**	**<0.001**	**<0.001**	**0.002**	**<0.001**	**<0.001**
**Salad Dressings**								
Regular (*n* = 69)	427.6 (11.7)	891.7 (30.1)	40.9 (1.4)	6.1 (0.3)	129.0 (3.7)	269.9 (9.9)	12.3 (0.4)	1.8 (0.1)
Modified (*n* = 88)	192.4 (7.4)	974.2 (26.1)	12.1 (1.0)	1.7 (0.2) ^5^	58.9 (2.2)	298.9 (8.1)	3.6 (0.3)	0.5 (0.1) ^5^
Average Difference ^2^	−226.2 (8.4)	75.8 (23.2)	−27.7 (1.1)	−4.2 (0.2)	−67.6 (2.6)	26.4 (7.5)	−8.4 (0.3)	−1.3 (0.1)
Percent Change ^3^	−52.9	8.5	−67.7	−68.9	−52.4	9.8	−68.3	−72.2
*p*-value ^4^	**<0.001**	**0.002**	**<0.001**	**<0.001**	**<0.001**	**0.001**	**<0.001**	**<0.001**
**Savory Snacks**								
Regular (*n* = 9)	528.6 (10.2)	792.9 (75.0)	33.5 (0.8)	8.3 (1.5)	155.6 (3.2)	236.7 (26.6)	9.9 (0.4)	2.6 (0.6)
Modified (*n* = 14)	395.5 (32.5)	650.6 (41.2)	14.8 (3.3)	4.2 (1.2)	96.1 (4.6)	171.4 (19.6)	3.3 (0.6)	0.9 (0.3)
Average Difference ^2^	−132.1 (34.6)	−80.5 (61.6)	−18.4 (3.6)	−4.9 (1.4)	−58.9 (4.1)	−46.4 (18.9)	−6.5 (0.7)	−1.9 (0.4)
Percent Change ^3^	−25.0	−10.2	−54.9	−59.0	−37.9	−19.6	−65.7	−73.1
*p*-value ^4^	**0.003**	0.230	**<0.001**	**0.005**	**<0.001**	0.034	**<0.001**	**0.001**
**Soups**								
Regular (*n* = 13)	70.8 (4.8)	429.2 (44.9)	3.6 (0.5)	0.9 (0.1)	145.4 (10.0)	833.8 (19.9)	7.0 (0.9)	1.8 (0.3)
Modified (*n* = 17)	46.0 (2.2)	335.8 (32.5)	1.4 (0.0) ^6^	0.4 (0.0) ^6^	88.8 (6.2)	612.9 (36.6)	2.5 (0.2)	0.7 (0.1)
Average Difference ^2^	−28.8 (3.0)	−141.1 (29.8)	−2.7 (0.4)	−0.6 (0.1)	−52.4 (4.8)	−229.4 (39.4)	−4.7 (0.6)	−1.1 (0.2)
Percent Change ^3^	−40.7	−32.9	−75.0	−66.7	−36.0	−27.5	−67.1	−61.1
*p*-value ^4^	**<0.001**	**<0.001**	**<0.001**	**0.001**	**<0.001**	**<0.001**	**<0.001**	**<0.001**
**25% Reduction in Labeled Fat**								
**Cheese**								
Regular (*n* = 73)	348.4 (9.0)	711.3 (38.7)	27.6 (0.9)	17.1 (0.6)	92.5 (2.4)	177.7 (7.8)	7.3 (0.2)	4.6 (0.1)
Modified (*n* = 86)	252.0 (8.7)	791.3 (44.0)	15.1 (0.9)	9.4 (0.6)	65.6 (2.1)	194.0 (9.0)	3.9 (0.2)	2.4 (0.1)
Average Difference ^2^	−87.7 (5.1)	82.4 (13)	−11.7 (0.6)	−7.3 (0.4)	−26.0 (1.4)	18.5 (3.6)	−3.4 (0.2)	−2.1 (0.1)
Percent Change ^3^	−25.2	11.6	−42.4	−42.7	−28.1	10.4	−46.6	−45.7
*p*-value ^4^	**<0.001**	**<0.001**	**<0.001**	**<0.001**	**<0.001**	**<0.001**	**<0.001**	**<0.001**
**Salad Dressings**								
Regular (*n* = 70)	423.8 (12.2)	889.6 (29.7)	40.5 (1.5)	6.0 (0.3)	127.9 (3.8)	269.4 (9.7)	12.2 (0.4)	1.8 (0.1)
Modified (*n* = 89)	191.3 (7.4)	973.3 (25.8)	12.0 (1.0)	1.7 (0.2) ^5^	58.7 (2.2)	299.8 (8.0)	3.6 (0.3)	0.5 (0.1) ^5^
Average Difference ^2^	−224.4 (8.5)	76.6 (23.0)	−27.5 (1.1)	−4.2 (0.2)	−67 (2.6)	27.7 (7.5)	−8.3 (0.3)	−1.3 (0.1)
Percent Change ^3^	−52.9	8.6	−67.9	−70.0	−52.4	10.3	−68.0	−72.2
*p*-value ^4^	**<0.001**	**0.001**	**<0.001**	**<0.001**	**<0.001**	**<0.001**	**<0.001**	**<0.001**
**Savory Snacks**								
Regular (*n* = 19)	536.1 (7.3)	705.8 (53.8)	33.3 (0.9)	7.2 (1.1)	155.3 (2.2)	205.8 (17.2)	9.7 (0.3)	2.2 (0.4)
Modified (*n* = 31)	414.9 (15.0)	649.5 (25.8)	13.9 (1.4)	2.8 (0.5)	112.7 (3.5)	181.5 (10.2)	3.6 (0.3)	0.7 (0.1)
Average Difference ^2^	−124.0 (15.9)	−22.6 (37.8)	−19.5 (1.7)	−4.3 (0.8)	−42.4 (3.6)	−13.4 (12.2)	−6 (0.4)	−1.4 (0.3)
Percent Change ^3^	−23.1	−3.2	−58.6	−59.7	−27.3	−6.5	−61.9	−63.6
*p*-value ^4^	**<0.001**	0.562	**<0.001**	**<0.001**	**<0.001**	0.290	**<0.001**	**<0.001**
**Soups**								
Regular (*n* = 19)	64.8 (4.4)	412.3 (37.7)	3.0 (0.5)	0.7 (0.1)	134.7 (8.0)	815.8 (18.4)	5.7 (0.8)	1.5 (0.2)
Modified (*n* = 24)	46.6 (1.9)	315.6 (29.8)	1.1 (0.1)	0.3 (0.0) ^6^	94.2 (5.1)	591.7 (32.8)	2.0 (0.2)	0.6 (0.1)
Average Difference ^2^	−21.3 (3.2)	−129.9 (23.1)	−2.2 (0.3)	−0.5 (0.1)	−38.8 (5.4)	−232.1 (34.9)	−3.9 (0.5)	−0.9 (0.1)
Percent Change ^3^	−32.9	−31.5	−73.3	−71.4	−28.8	−28.5	−68.4	−60.0
*p*-value ^4^	**<0.001**	**<0.001**	**<0.001**	**<0.001**	**<0.001**	**<0.001**	**<0.001**	**<0.001**

SE = Standard Error; Sat = Saturated. Boldface signifies statistical significance (*p* < 0.00625), Bonferroni adjustment for multiple comparisons of *p* < 0.05. ^1^ Some products may have both 25% less calories and 25% less fat; ^2^ The difference in nutrient content for each match was calculated and averaged over all matched pairs (modified-regular). The number of matched pairs for each analysis is equal to the number of modified products. One regular food may have one or more modified food counterparts; ^3^ Percent Change was calculated using the following formula: $\frac{{\text{Mean}}_{\text{Difference}}}{{\text{Mean}}_{\text{Reg}}}*100$; ^4^ *p*-values were determined by paired *t*-tests between regular and modified foods. Statistical significance was defined as *p*-value < 0.00625; ^5^ One modified salad dressing product was excluded due to lack of information on saturated fat content; *N* = 87 for products with 25% reduction in calories; *N* = 88 for products with 25% reduction in fat; ^6^ SE < 0.05.

## Supplementary Materials

The following are available online at http://www.mdpi.com/2072-6643/8/8/511/s1, Figure S1: Flow diagram depicting selection process for food categories, Figure S2: Websites used to collect nutrition data for regular and modified foods identified in the top 10 brands within the four food categories, Figure S3: Flow diagram depicting derivation of product nutrition information, Table S1: Serving size (g) by food category for regular and modified foods, Table S2: Mean calorie, sodium, total fat, and saturated fat by food category for regular and modified foods, where each regular food product was randomly matched to one modified counterpart.
