# Supplementary material for: Do Lower Calorie or Lower Fat Foods Have More Sodium Than Their Regular Counterparts?
Source: Nutrients. 2016 Aug 19;8(8):511. doi: 10.3390/nu8080511 (PMC4997424; doi:10.3390/nu8080511)
Supplement: Supplementary file 1 [file nutrients-08-00511-s001.docx]

Supplementary Materials: Do Lower Calorie or Lower Fat Foods Have More Sodium Than Their Regular Counterparts?

Katherine A. John, Joyce Maalouf, Christina B. Barsness, Keming Yuan, Mary E. Cogswell and Janelle P. Gunn

**Figure S1.** Flow diagram depicting selection process for food categories.


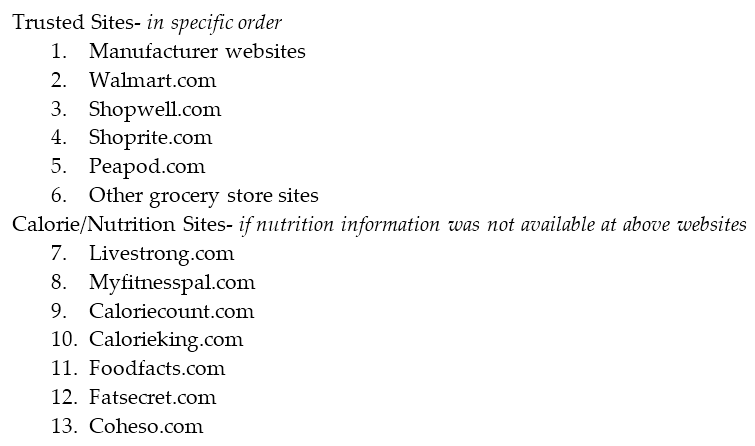


**Figure S2.** Websites used to identify products within the top 10 brands and to collect nutrition data on these regular and modified foods identified in the top 10 brands within the four food categories.


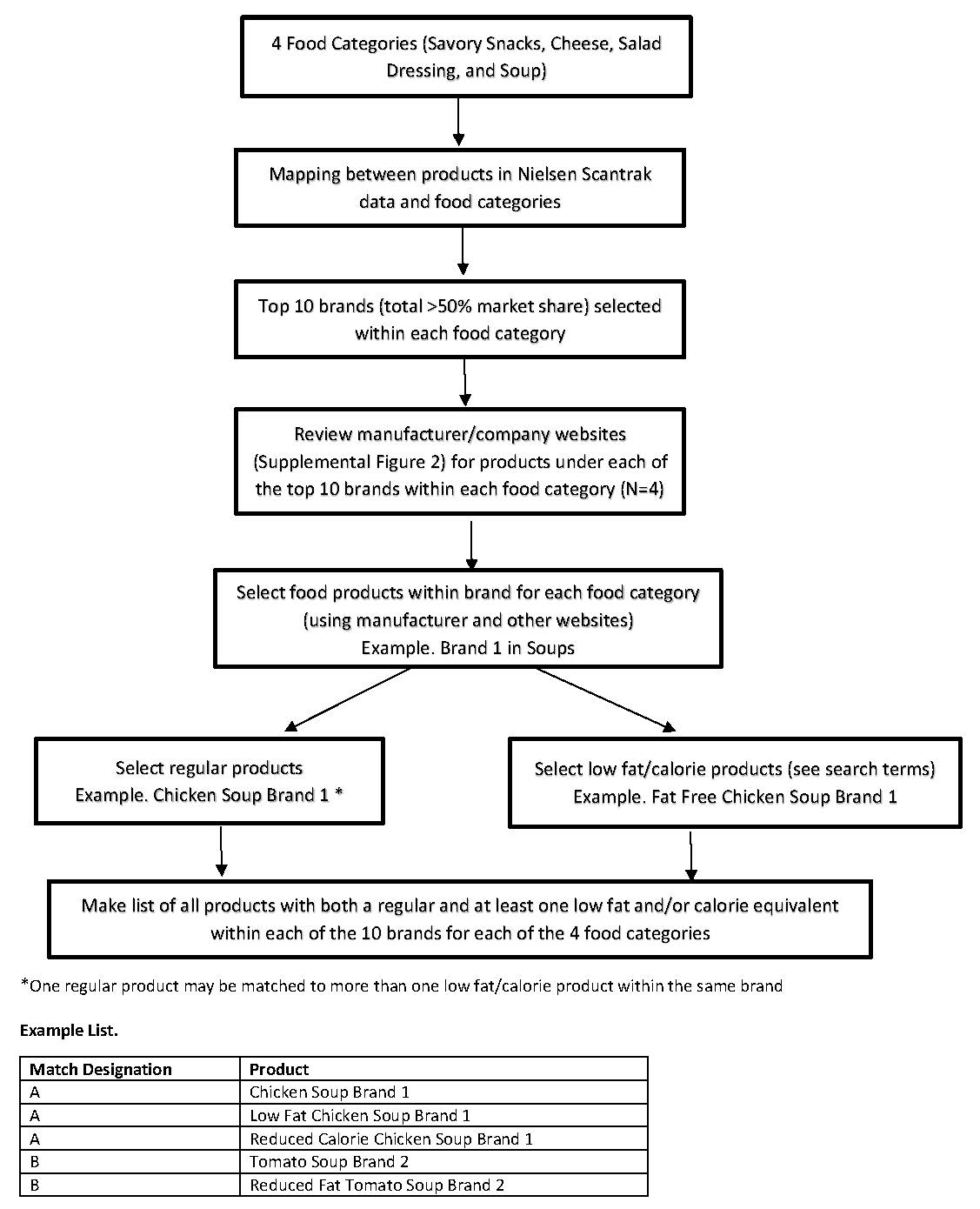


**Figure S3.** Flow diagram depicting derivation of product nutrition information.

**Table S1.** Serving size (g) by food category for regular and modified foods.

| **Food Category** | **Serving Size (g)** | |
| --- | --- | --- |
|  | **Mean (SE)** | **Range** |
| **Cheese** |  |  |
| Regular (*n* = 83) | 29.1 (2.3) | 5–196 |
| Modified (*n* = 105) | 30.3 (2.0) | 5–196 |
| **Salad Dressings** |  |  |
| Regular (*n* = 71) | 30.3 (0.4) | 15–44 |
| Modified (*n* = 90) | 30.9 (0.3) | 15–42.5 |
| **Savory Snacks** |  |  |
| Regular (*n* = 23) | 29.2 (0.5) | 28–35 |
| Modified (*n* = 44) | 29.3 (1.0) | 18–45 |
| **Soups** |  |  |
| Regular (*n* = 37) | 209.5 (9.2) | 120–301 |
| Modified (*n* = 44) | 208.0 (8.9) | 120–301 |

SE = Standard Error.

**Table S2.** Mean calorie, sodium, total fat, and saturated fat by food category for regular and modified foods, where each regular food product was randomly matched to only one modified counterpart.

|  | **Per 100 g of Food** | | | | **Per Labeled Serving Size** | | | |
| --- | --- | --- | --- | --- | --- | --- | --- | --- |
|  | **Mean (SE) Calories (kcal)** | **Mean (SE) Sodium (mg)** | **Mean (SE) Total Fat (g)** | **Mean (SE) Sat. Fat (g)** | **Mean (SE) Calories (kcal)** | **Mean (SE) Sodium (mg)** | **Mean (SE) Total Fat (g)** | **Mean (SE) Sat. Fat (g)** |
| **Cheese** | | | | | | | | |
| Regular (*n* = 83) | 347.3 (8.1) | 698.6 (34.8) | 27.4 (0.8) | 17.0 (0.5) | 91.3 (2.2) | 174.3 (7.1) | 7.2 (0.2) | 4.5 (0.1) |
| Modified (*n* = 83) | 274.1 (7.6) | 769.2 (39.3) | 17.7 (0.7) | 10.9 (0.5) | 72.8 (2.2) | 195.1 (8.3) | 4.7 (0.2) | 2.9 (0.1) |
| Average Difference ^a^ | −73.2 (4.7) | 70.5 (12.8) | −9.7 (0.6) | −6.1 (0.4) | −18.6 (2.0) | 20.8 (4.0) | −2.5 (0.2) | −1.6 (0.1) |
| Percent Change ^b^ | −21.1 | 10.1 | −35.4 | −35.9 | −20.4 | 11.9 | −34.7 | −35.6 |
| *p*-value ^c^ | **<0.001** | **<0.001** | **<0.001** | **<0.001** | **<0.001** | **<0.001** | **<0.001** | **<0.001** |
| **Salad Dressings** | | | | | | | | |
| Regular (*n* = 71) | 422.3 (12.1) | 889.8 (29.3) | 40.3 (1.5) | 6.0 (0.3) | 127.5 (3.8) | 269.6 (9.6) | 12.1 (0.4) | 1.8 (0.1) |
| Modified (*n* = 71) | 192.7 (7.7) | 956.9 (29.4) | 12.2 (1.1) | 1.8 (0.2) | 59.5 (2.4) | 296.1 (9.4) | 3.7 (0.3) | 0.6 (0.1) |
| Average Difference ^a^ | −229.6 (10.0) | 67.1 (26.0) | −28.1 (1.3) | −4.3 (0.3) | −68.0 (3.2) | 26.5 (8.9) | −8.4 (0.4) | −1.3 (0.1) |
| Percent Change ^b^ | −54.4 | 7.5 | −69.7 | −71.7 | −53.3 | 9.8 | −69.4 | −72.2 |
| *p*-value ^c^ | **<0.001** | 0.012 | **<0.001** | **<0.001** | **<0.001** | **0.004** | **<0.001** | **<0.001** |
| **Savory Snacks** | | | | | | | | |
| Regular (*n* = 23) | 533.9 (6.4) | 699.5 (45.4) | 33.0 (0.8) | 7.8 (1.0) | 155.2 (1.9) | 204.8 (14.6) | 9.7 (0.3) | 2.4 (0.4) |
| Modified (*n* = 23) | 452.2 (16.1) | 629.9 (30.0) | 19.0 (2.0) | 4.8 (1.0) | 133.5 (6.6) | 189.1 (13.0) | 5.7 (0.7) | 1.5 (0.4) |
| Average Difference ^a^ | −81.6 (17.2) | −69.6 (46.8) | −14.0 (2.3) | −3.0 (0.8) | −21.7 (6.2) | −15.7 (15.5) | −4.0 (0.7) | −0.9 (0.3) |
| Percent Change ^b^ | −15.3 | −10.0 | −42.4 | −38.5 | −14.0 | −7.7 | −41.2 | −37.5 |
| *p*-value ^c^ | **0.001** | 0.159 | **<0.001** | **0.002** | **0.002** | 0.335 | **<0.001** | **0.006** |
| **Soups** | | | | | | | | |
| Regular (*n* = 37) | 62.5 (3.3) | 428.7 (29.2) | 2.0 (0.3) | 0.5 (0.1) | 124.3 (5.6) | 805.7 (15.7) | 3.9 (0.5) | 1.0 (0.1) |
| Modified (*n* = 37) | 50.1 (3.0) | 265.6 (18.4) | 1.0 (0.1) | 0.3 (0.0) ^d^ | 102.2 (4.8) | 528.1 (26.0) | 2.0 (0.2) | 0.6 (0.1) |
| Average Difference ^a^ | −12.4 (2.5) | −163.1 (21.3) | −1.0 (0.2) | −0.2 (0.1) | −22.2 (4.2) | −277.6 (29.3) | −1.9 (0.4) | −0.5 (0.1) |
| Percent Change ^b^ | −19.8 | −38.1 | −50.0 | −40.0 | −17.9 | −34.5 | −48.7 | −50.0 |
| *p*-value ^c^ | **<0.001** | **<0.001** | **<0.001** | **0.002** | **<0.001** | **<0.001** | **<0.001** | **0.001** |

SE = Standard Error; Sat = Saturated. Boldface signifies statistical significance (*p* < 0.00625, Bonferroni adjustment for multiple comparisons of *p* < 0.05). ^a^ The difference in nutrient content for each match was calculated and averaged over all matched pairs (modified-regular). The number of matched pairs for each analysis is equal to the number of modified products. ^b^ Percent Change was calculated using the following formula: $\frac{\mathrm{Mean}_{\mathrm{Difference}}}{\mathrm{Mean}_{\mathrm{Reg}}}*100$. ^c^ *p*-values were determined by paired *t*-tests between regular and modified foods. Statistical significance is defined as *p*-value < 0.00625. ^d^ SE < 0.05.
